# Universal Energy Solution for Triboelectric Sensors Toward the 5G Era and Internet of Things

**DOI:** 10.1002/advs.202302009

**Published:** 2023-05-28

**Authors:** Haiyang Wen, Xiya Yang, Ruiyuan Huang, Duo Zheng, Jingbo Yuan, Hongxin Hong, Jialong Duan, Yunlong Zi, Qunwei Tang

**Affiliations:** ^1^ Institute of New Energy Technology College of Information Science and Technology Jinan University Guangzhou 510632 China; ^2^ School of Physics and Optoelectronics South China University of Technology Guangzhou 510641 China; ^3^ Institute of Carbon Neutrality College of Chemical and Biological Engineering Shandong University of Science and Technology Qingdao 266590 China; ^4^ Thrust of Sustainable Energy and Environment The Hong Kong University of Science and Technology (Guangzhou) Nansha Guangzhou, Guangdong 511400 China

**Keywords:** electronic circuit design, Internet of Things, power management, smart switching system, triboelectric sensors

## Abstract

The launching of 5G technology provides excellent opportunity for the prosperous development of Internet of Things (IoT) devices and intelligent wireless sensor nodes. However, deploying of tremendous wireless sensor nodes network presents a great challenge to sustainable power supply and self‐powered active sensing. Triboelectric nanogenerator (TENG) has shown great capability for powering wireless sensors and work as self‐powered sensors since its discovery in 2012. Nevertheless, its inherent property of large internal impedance and pulsed “high‐voltage and low‐current” output characteristic seriously limit its direct application as stable power supply. Herein, a generic triboelectric sensor module (TSM) is developed toward managing the high output of TENG into signals that can be directly utilized by commercial electronics. Finally, an IoT‐based smart switching system is realized by integrating the TSM with a typical vertical contact–separation mode TENG and microcontroller, which is able to monitor the real‐time appliance status and location information. Such design of a universal energy solution for triboelectric sensors is applicable for managing and normalizing the wide output range generated from various working modes of TENGs and suitable for facile integration with IoT platform, representing a significant step toward scaling up TENG applications in future smart sensing.

## Graphical abstract

A generic triboelectric sensor module toward managing the high output of triboelectric nanogenerator into signals that can be directly utilized by commercial electronics is developed with the merits of highly integrated, small‐dimension, and high‐sensitivity, which endows seamless integration with Internet of Things platform for large‐scale application as smart sensing network.

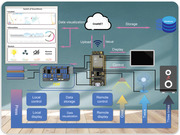

## 1 Introduction

IoT era, billions of smart sensing devices will experience fast wireless communication,^[^ ^1^ ^]^ and one of the open challenge is the power supply for the decentralized IoT devices’ “edge” computing and real‐time sensors that could be hard to be sustained by centralized power grid.^[^ ^2^ ^]^ Although traditional sensor technology has achieved mature industrial production and more accurate information collection functions, however, its dimension, cost, and dependence on battery power supply also hindered their development into a sensor network.^[^ ^3^ ^]^ On the other hand, batteries might not be the best solution for the IoT sensing network due to their limited lifetime, size, and potential environmental problems.^[^ ^4^ ^]^ Apart from this, wide distribution of the sensors and high maintenance costs make batteries an insufficient solution, especially for remote and inaccessible areas.^[^ ^5^ ^]^ Thus, the pursue for renewable energy sources for large‐scale usage has become an alternative solution to solve the enormous energy demands and there is an urgent requirement for active self‐powered sensors with high‐sensitivity and accurate monitoring ability to collect real‐time information from the gigantic IoT sensor network.^[^ ^6^ ^]^

Triboelectric nanogenerator (TENG), since its discovery in 2012, has been proved to be a promising energy harvesting technology, especially for scavenging low‐frequency mechanical energy, owing to their flexibility in design, easy manufacture, excellent signal to noise ratio, and broad material applicability.^[^ ^7^, ^8^, ^9^, ^10^, ^11^, ^12^, ^13^, ^14^ ^]^ The self‐powered feature of TENG effectively solves the problems of short‐life and environmental pollution of traditional active sensors powered by batteries, which completely fits the need for decentralized IoT devices and the underlying signal acquisition in the IoT era.^[^ ^15^ ^]^ Moreover, the features of small dimension and lightweight of TENG make it easily to be integrated with IoT network as self‐powered sensors in the fields of smart wearable devices,^[^ ^16^, ^17^ ^]^ smart healthcare,^[^ ^18^, ^19^ ^]^ smart home,^[^ ^20^, ^21^ ^]^ smart transportation,^[^ ^22^ ^]^ and smart city.^[^ ^23^ ^]^ As the significant interface between the TENG and load electronics, power management circuit (PMC) is essential and plays important role in the voltage and impedance conversion for efficient energy transmission, supply, and storage. The most widely adopted PMC at present for triboelectric sensors is switching power management, which can be categorized into mechanical and electronic switches based on the switch types. Based on the periodic movement of TENG, the switching frequency can be controlled once or twice the output frequency of the TENG electric output signals, enabling the change of continuous energy release into instantaneous discharge to enhance the instantaneous power output and energy output.^[^ ^24^, ^25^, ^26^, ^27^, ^28^ ^]^ However, the operation mode of this kind of switch is seriously limited by the working mode of TENG and suffer from low universality, since the switch can only operate at a specific frequency or voltage amplitude, significantly affecting the stable and high‐efficient electrical output. On the other hand, the PMC based on electronic switch is more flexible and adaptable, which can accurately track the peak of TENG voltage and adapt to the changes of external excitation.^[^ ^29^, ^30^, ^31^, ^32^, ^33^ ^]^ Nevertheless, most of the electronic switch based PMC relies on an external power supply, and for the semiconductor‐based electronic switches, which cannot achieve absolute isolation in an open‐circuit state.^[^ ^34^ ^]^ Meanwhile, some PMCs based on integrated operational amplifiers with non‐biased static voltage characteristic will discard the signal characteristic of the negative half‐axis waveform of the TENG output voltage,^[^ ^35^ ^]^ or add another set of power supply to retain the negative half‐axis waveform, which could increase the power consumption of the system,^[^ ^36^ ^]^ coupled with the intrinsic high‐voltage output property, electrostatic breakdown can be easily occurred thus bringing damage to a transistor based PMC.^[^ ^37^ ^]^ In addition to the characteristic of voltage bias, the characteristics of adjustable input impedance, filtering ability, analog output, and digital conversion are compared with peers’ works as given in Table S1, Supporting Information. In a word, development of a universal energy solution to realize the transformation from micro to large energy and apply to various working modes of TENG will be the key toward large‐scale application of TENG energy supply in the future.

Herein, we developed a generic triboelectric sensor module (TSM) based on the concept of impedance matching and signal filtering, which effectively solves the problem of high‐voltage output of TENG and converts the output into a stable high and low‐level that can be directly read by commercial electronics. Moreover, on the basis of low‐frequency active sensing circuit, the filtering performance can be further improved to withstand more complex signal interference and stabilize the signal output performances. Finally, by integrating the TSM with a typical vertical contact–separation (CS) mode TENG and microcontroller, an IoT‐based smart switching system is realized for monitoring the real‐time appliance status and location information. Such design of a universal energy solution for triboelectric sensors is applicable for standardizing and normalizing the outputs generated from various working modes of TENGs and suitable for facile integration with IoT platform, which could be a key technology to promote the development for IoT and 5G communication.

## 2 Results and Discussion

### 2.1 Working Characteristics of TSM and Performance Optimization

**Figure 1:**
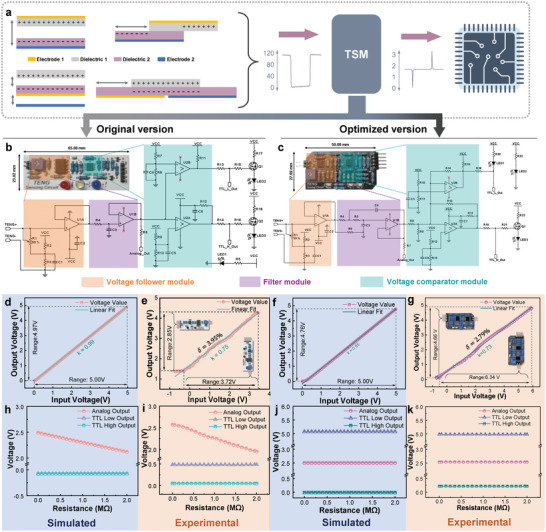
a) Workflow of the TSM connected with the general four modes of TENGs and commercial sensing devices. Circuit diagram comparison of the b) original and c) optimized TSM including the voltage follower, filter, and voltage comparator functional circuit modules. Full‐scale output simulation and experimental results of the d,e) original and f,g) optimized TSM, respectively. Simulation and experimental results for the dependence of the output port voltage on input resistance (potentiometer) of the h,i) original and j,k) optimized TSM, respectively.

The original open‐circuit voltage (*V*_OC_) generated by various modes of TENGs requires signal conditioning and power management to be better recognized and read by subsequent commercial electronic devices for sensing applications as schematized in **Figure** **1**a. The circuit diagrams of the original and optimized TSM are demonstrated in Figure 1b and 1c, respectively. The main functional modules of TSM can be divided into three modules: the voltage follower module including the direct‐current (DC) bias and the voltage follower circuit (orange area); the active low‐pass filter module with first‐order for the original TSM and second‐order for the optimized TSM (purple area); a voltage comparator module (green area) including an upper‐limit and a lower‐limit voltage comparison circuit as well as an indicator light circuit (the optimized TSM has a hysteresis comparator circuit). The overall working process for the TSM can be described as the alternating‐current (AC) output signals generated by the TENG transmit first to the TSM, and then pass through a DC bias circuit, which is mounted on a stable DC voltage to prevent cut‐off distortion. Since the input impedance of the integrated operational amplifier is infinite under ideal condition (which could be as high as 3000 GΩ in practical situation) and the output impedance is 0 Ω, the input impedance of the TSM depends on the current value of the potentiometer by connecting the input of TENG to the non‐inverting input of the integrated operational amplifier, and then with the potentiometer in parallel connection. According to the Ohm's law, the impedance matching can be realized by adjusting the resistance of the potentiometer to obtain a suitable voltage input for the voltage follower module. The signal undergoes impedance conversion through the integrated operational amplifier to reduce the output impedance and enable it to have a better ability to drive the load electronics. Furthermore, active filter circuit is utilized to filter high‐frequency clutter toward obtaining stable low‐frequency signals. Finally, the output analog signal is passed into the voltage comparison module to compare with the upper and lower limit voltage to yield a stable TTL level that matches the analog signal for processing by the digitizing circuit; the flashing of the indicator light demonstrates the signal output.

The static characteristics determine and form the static operating point of the DC circuit under different conditions, and the static operating point serves as the basis for carrying dynamic signals that not only determines the distortion of the circuit, but also affects the step‐down amplitude of voltage, input resistance, and other dynamic parameters.^[^ ^38^ ^]^ Hence, static characteristics of TSM are essential to be investigated through both simulation and experimental approach. It can be seen from Figure 1d–g, the full‐scale output simulation results for the original and optimized TSM are similar, however, the experimental results of the optimized TSM behaves better with a higher voltage input of 6.34 V and output of 4.66 V as well as a higher linearity *δ* of 2.79%. The dependence of the output port voltage on input resistance for the optimized TSM (Figure 1j,k) demonstrates relatively stable static output compared with the original version on Figure 1h,i in terms of both simulation and experimental results. Due to the insufficient input resistance of the integrated operational amplifier, the voltage of analog output port will decrease with the increase of the resistance of potentiometer in the original TSM, thus affecting the voltage output and voltage amplitude discrimination of the subsequent circuit.

**Figure 2:**
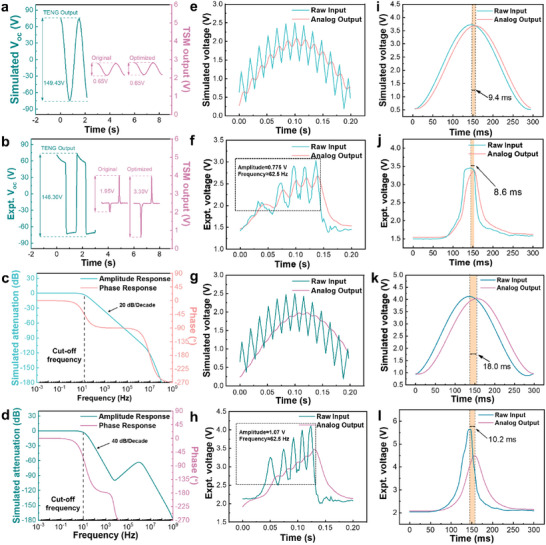
a,b) Acquisition ability for capturing TENG output voltage with potentiometer resistance of 2 MΩ. c,d) Amplitude‐frequency and phase‐frequency characteristic curve of the frequency response. e–h) Triangular interference wave loading on signals. i–l) Response time.

The quality and delay of the waveform during signal transmission are mainly determined by dynamic characteristics,^[^ ^39^ ^]^ which are further carried out on the DC output under the same static conditions with power supply of 5 V and input resistance potentiometer of 2 MΩ. The dynamic parameters including the signal acquisition ability, response time, filtering capability, voltage amplitude discrimination ability, and distortion condition are systematically examined. The acquisition ability of TSM for capturing TENG output voltage is tested with a sine wave voltage signal with amplitude and frequency of 75 V and 0.67 Hz, respectively, and a capacitor of 500 pF is connected in series. After the signal passes through the two versions TSM, the simulated output voltage of the analog end is almost the same as demonstrated in **Figure** **2**a. In the running of the actual circuit using the TENG output, the voltage amplitude obtained by the optimized TSM is higher and more complete compared to that obtained by the original version as shown in Figure 2b, the amplitude difference can be attributed to the differentiation in the signal amplitude acquisition capability of the two version TSM.

As a significant factor affecting the signal‐to‐noise ratio in TENG sensing circuit, the filtering capability directly determines the quality of the waveform of output analog signals.^[^ ^40^ ^]^ Hence, the frequency response characteristics including the attenuation and phase shift of the two version TSM are simulated as demonstrated in Figure 2c,d. Although the original TSM has better phase shift characteristic, while the high frequency attenuation is only half of the optimized TSM (20 dB/decade), thus greatly reducing the filtering effect. In order to show the filtering characteristics more clearly, we evaluate the simulated and actual TSM by adding a 60 Hz triangular interference wave as shown in Figure 2e–h, the raw input at around 60 Hz frequency and 0.7–1.0 V amplitude noise indicated that the optimized TSM behaved a smoother signal processing ability (Figure 2g,h) than the original TSM (Figure 2e,f) in analog output port. For comparison, rectangular and trapezoidal interference waves are also loaded to test the signal processing behavior as demonstrated in Figure S2, Supporting Information. The number of components in the circuit also determine the response time of the two version TSM. As shown in Figure 2i,k, the simulated response time for the optimized TSM is around 18.0 ms, which is almost twofold delayed compared to that of the original TSM of 9.4 ms. For the experimental results on Figure 2j–l, the response time for the optimized TSM is 10.2 ms, which is also slightly higher than that of the original version of 8.6 ms.

**Figure 3:**
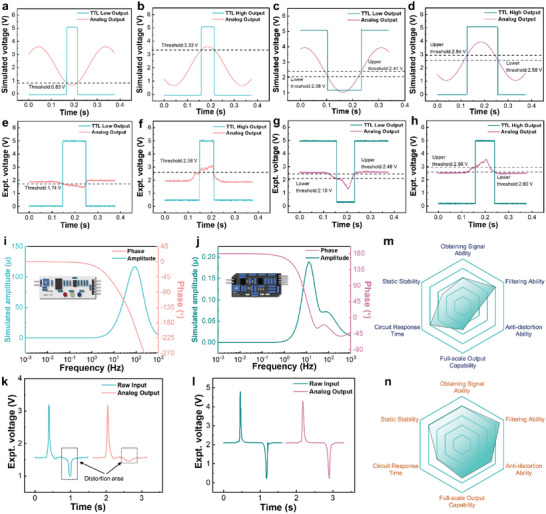
Simulated and experimental measurement results for a–h) the lower and upper threshold voltage. i,j) Amplitude and phase second harmonic distortion simulation results. k,l) Waveform distortion and m,n) overall performance evaluation using radar chart for the original and optimized TSM.

As a form of signal transmission that complements analog signal, digital signals have been widely used in the field of modern signal processing.^[^ ^41^ ^]^ Therefore, a stable and reliable digital signal not only determines the stability received by the commercial electronic devices, but also affects the accuracy of the received information. The simulated analog data of the threshold voltage of the voltage comparator for the two versions TSM are illustrated in **Figure** **3**a–d. Under the condition of 5 V supply voltage, the simulated values of the lower and upper threshold voltages of the original TSM are 0.83 and 3.33 V, respectively (Figure 3a,b), while the lower and upper threshold voltages of the optimized TSM are 2.06 V/2.41 V and 2.94 V/2.58 V in Figure 3c,d. The original TSM has two single‐limit comparators, and there is only one voltage threshold when comparing the upper and lower limit voltages, while the optimized TSM has two hysteresis comparators, and there are two thresholds when comparing the upper and lower limit voltages, respectively. This indicates that when comparing the analog voltage, unlike a single‐limit comparator in original TSM flips, once the voltage threshold is reached, the digital output port of the optimized TSM will invert signal of the static output by the time that the analog voltage of the signal reaches the first threshold, and it will flip back to the static voltage when it reaches the second voltage threshold, which reduces the possibility of multiple outputs of one signal to a certain extent. The corresponding experimental results of the two version TSM show that the lower and upper threshold voltages of the original TSM are 1.74 and 2.58 V, respectively, in Figure 3e,f, and the lower and upper threshold voltages of the optimized TSM are 2.10 V/2.46 V and 2.96 V/2.60 V, respectively, in Figure 3g,h, which exhibits similar trend in voltage thresholds and indicates that the optimized TSM has better anti‐interference ability, signal stability, and signal delay ability during voltage judgment.

In addition to the threshold voltage, distortion in the active sensing circuit is also an important factor that determines whether the output analog signal has a high degree of signal restoration.^[^ ^42^ ^]^ The simulated second total harmonic distortion curves for the two versions TSM are demonstrated in Figure 3i,j, it can be seen that the second total harmonic distortion of the original TSM is three orders of magnitude higher than that of the optimized TSM, indicating the anti‐distortion ability for the optimized TSM is better compared to the original version. Furthermore, we applied the TENG output as the input signal to evaluate and compare the actual circuit performances for the two versions TSM, the analog output of the optimized TSM on Figure 3l exhibited a high degree of signal restoration compared to that on Figure 3k, demonstrating the excellent anti‐distortion capability of the optimized TSM. Overall, radar chart is adopted to evaluate the comprehensive working performances of the two versions TSM in the aspect of static and dynamic characteristics, including the static stability, signal acquisition, filtering capability, anti‐distortion ability, full‐scale output, and response time as illustrated in Figure 3m,n and Table S2, Supporting Information. Moreover, the voltage output and temperature stability of each port for the optimized TSM were measured for 5 days at every 2 h as demonstrated in Figure S3, Supporting Information. Considering the overall performances and working stability, the optimized TSM is confirmed to be a universal energy solution for triboelectric sensors, which is selected for further integrating with various modes of TENG for subsequent smart sensing applications.

### 2.2 Integration of TENG with TSM and Comprehensive Performance Evaluation

**Figure 4:**
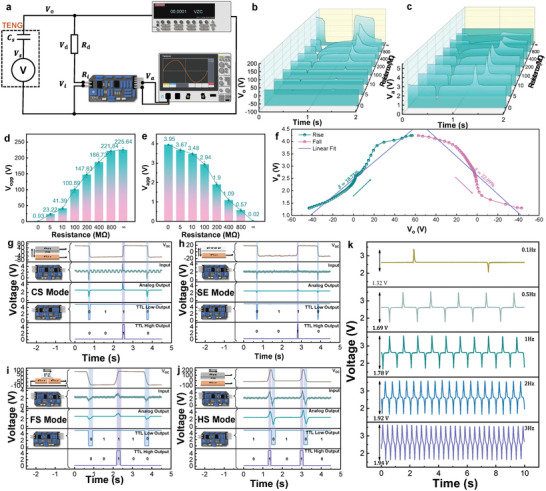
a) Circuit test diagram of the correspondence between the original output of TENG and the signal output from the optimized TSM. b,c) Corresponding waveform diagrams of the output of TENG and the TSM by changing the divider resistance. d,e) Peak‐to‐peak voltage output correspondence between the TENG (*V*_opp_) and TSM (*V*_app_) as resistance changes in the circuit. f) Corresponding linearity relationship between the *V*_o_ and *V*_a_ with *R*_d_ of 100 MΩ. g–j) Output waveforms of TENG and each port output of the optimized TSM for the four working modes of TENG, respectively. k) Waveform comparison for the frequency dependence of voltage output of the optimized TSM using vertical CS mode.

In order to investigate the corresponding relationship between the original output voltage (*V*_o_) of TENG and the signal output form the TSM, we applied the circuit as indicated in **Figure** **4**a by connecting the TENG with TSM through a series divider resistor *R*_d_. Keithley 6514 is utilized to measure the original output of TENG (*V*_o_), including the voltage of the divider resistor *R*_d_ (*V*_d_) and the input port voltage of the TSM (*V*_i_), due to the characteristics of resistance hindering the flow of electrons, the resistor *R*_d_ with large‐value is connected with the test circuit as a series resistor to increase the input impedance of the entire test circuit, so that the 6514 electrometer can capture a large voltage value and waveform that is close to the open‐circuit output of TENG. An oscilloscope is used to measure the analog output voltage from the TSM (*V*_a_). Herein, Ohm's law is used to calculate the voltage division value of each part. Under the specific working frequency of TENG, the equivalent impedance *Z*_cs_ can be calculated by Equation (3) assuming the value of the equivalent small capacitance inside the TENG does not change, and further convert it into a pure resistance equivalent circuit to facilitate the calculation of the above parameters as shown in the Figure S4, Supporting Information. The original output of TENG (*V*_o_) is significantly affected by the value of *R*_d_ as given by the Equation (1) below:

(1) $$V_{o}=V_{s}\times \frac{R_{d}+R_{i}}{Z_{\mathrm{cs}}+R_{d}+R_{i}}$$

where *V*_o_ is the output voltage of TENG, *V*_s_ is the internal voltage generated by TENG, *R*_d_ is the series divider resistor, *R*_i_ is the input resistance of the circuit, and *Z*_cs_ is the equivalent internal impedance of TENG. According to this equation, the value of *V*_o_ is proportional to the *R*_d_ resistance. As the value of *R*_d_ increases, *V*_o_ is closer to the open‐circuit output of TENG (*V*_OC_). In this circuit, the series divider resistor *R*_d_ and the internal impedance *Z*_cs_ can be regarded as the equivalent internal impedance of TENG, and its voltage division can be deduced according to the following Equation (2), which is inversely proportional to the value of *R*_d_.

(2) $$V_{i}=V_{s}\times \frac{R_{i}}{Z_{\mathrm{cs}}+R_{d}+R_{i}}$$

where *V*_i_ is the input voltage across the circuit. The internal impedance *Z*_cs_ can be obtained by Equation (3) given below:^[^ ^43^ ^]^

(3) $$Z_{\mathrm{cs}}=\frac{1}{2\pi fC_{s}}$$

where *f* and *C*_s_ are the natural frequency and internal equivalent capacitance of TENG, respectively. The resistance (*R*_d_) dependence of the original output of TENG (*V*_o_) and the voltage output from the TSM (*V*_a_) are demonstrated in Figures 4b and 4c, respectively. It can be observed that the two waveforms demonstrating complementary trends as the divider resistor (*R*_d_) increased from 0 to infinity. In addition, the peak‐to‐peak voltage output correspondence between the TENG (*V*_opp_) and TSM (*V*_app_) demonstrates a linearity with *R*_d_ as shown in Figure 4d,e. When *R*_d_ continuously improved above 100 MΩ, a waveform offset is clearly formed as shown in Figure S5, Supporting Information, hence, *R*_d_ of 100 MΩ is selected owing to its smaller waveform offset. Finally, a rising linearity *δ* of 18.7% from −45 to 60 V and a falling linearity of 22.09% from 60 to −45 V are registered as displayed in Figure 4f and Figure S5, Supporting Information, respectively, indicating the good linear relationship between the input *V*_o_ and output *V*_a_.

After investigating the correspondence between the TENG output and the signal output from the TSM, we applied the TSM to integrate with the four common working modes of TENG, including the vertical CS Mode, single‐electrode mode, freestanding mode, and horizontal sliding mode as shown in Figure 4g–j,^[^ ^44^ ^]^ to further explore its universal applicability. Polytetrafluoroethylene and copper (Cu) are selected as the negative and positive triboelectric materials for all the four working modes. Through observing the waveforms of *V*_oc_ of TENG, input port of TENG, analog output port, TTL low output port, and TTL high output port to assess the capability to process the analog signals and various output signals for the optimized TSM. The output waveforms generated from the four working modes of TENG are uniformly converted into sine signals (Figure 4g–i), while the horizontal sliding mode produces two unique waveforms with two waveform transitions as illustrated in Figure 4j. Moreover, the TENG movement of one cycle can be converted into two complete sine waves and two sets of high and low digital level outputs, indicating that the TENG outputs can be normalized under various working modes and demonstrates good followability and fast response. Finally, the frequency dependence of the analog output of the optimized TSM is carried out using the vertical CS Mode. It can be observed on Figure 4k and Figure S6, Supporting Information, that the *V*_oc_ of the TSM has no obvious distortion and waveform deformation except the amplitude due to the capacitive effect changing with frequency. According to Equation (3), the value of internal impedance *Z*_cs_ of TENG will decrease with the increased motion frequency of TENG, and the voltage *V*_i_ obtained by the device will increase based on Equation (4), which is consistent with the experimental results. Theoretically, assuming the operating frequency of 1 Hz and the equivalent internal capacitance and minimum input impedance of TENG are 1 nF and 100 kΩ, respectively, the as‐calculated maximum acceptable input voltage can reach around 8 KV according to Equations (3) and (4), which is universally applicable to various types of TENG.

(4) $$V_{i}=V_{\mathrm{oc}}\times \frac{R_{i}}{Z_{\mathrm{cs}}+R_{i}}$$

### 2.3 Demonstration of the Smart Switching System

**Figure 5:**
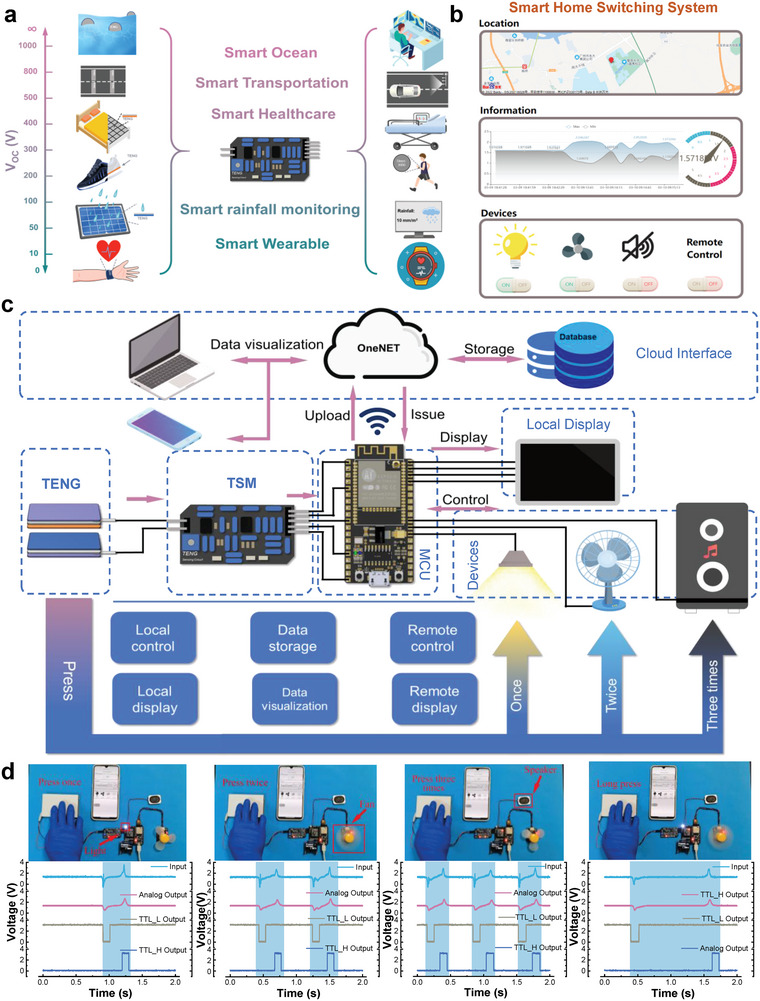
a) Potential application scenarios by applying the TSM for triboelectric sensors. b) Cloud interactive interface of the smart switching system. c) Schematic diagram of the smart switching system composing of a vertical CS Mode TENG, the TSM, MCU, local display device, other household devices, and human–computer interaction interface of cloud data visualization. d) Physical diagram demonstration of four control methods for different peripheral control and corresponding voltage waveforms produced from various TSM ports.

With the rapid development of IoT technology, sensors, as the core components for sensing external signals, have become the most indispensable nodes for information exchange between devices and control terminals in the IoT.^[^ ^45^, ^46^ ^]^ With the characteristics of overall perception, reliable transmission, and intelligent processing, IoT has been widely applied in modern smart systems, such as smart ocean,^[^ ^47^ ^]^ smart transportation,^[^ ^48^ ^]^ smart wearable,^[^ ^49^ ^]^ and smart healthcare^[^ ^50^ ^]^ as shown in **Figure** **5**a. Here, an IoT‐based smart switching system is built‐up by integrating the TSM with a typical vertical CS Mode TENG and microcontroller (Figure 5b,c), which is able to monitor the real‐time appliance status and location information toward achieving a comprehensive monitoring of the state of people at home and ensure their security as well as the appliances safety. Once an external mechanical incentive is transmitted to TENG, a distinguishable and stable voltage signal is generated and read by the MCU after being processed by the TSM. The continuous analog signal and a set of different digital signals in four control methods for different peripheral control and corresponding voltage waveforms generated from various TSM ports are demonstrated in Figure 5d and Video S1, Supporting Information, it can be seen that the external appliances can be controlled individually or synchronously. The real‐time appliance status, location information, and the voltage value on analog output port of TSM can be read and displayed on screen for human interface. Meanwhile, the above information is further transmitted to the OneNET IoT platform for cloud storage through wireless fidelity transmission, and a demonstration of remote control of the appliance switch can be found in Video S2, Supporting Information.

## 3 Conclusions

In conclusion, a universal energy solution for managing and normalizing the power output of TENG is proposed and a generic TSM is developed and successfully fabricated toward processing the output of TENG into signals that can be directly read and recognized by commercial electronic microprocessor. After systematically investigating the static and dynamic characteristics including the voltage acquisition capability, circuit response time, filtering capability, anti‐distortion capability, and voltage comparison capability, an optimized TSM with supply voltage ranging from 2.7 to 16 V corresponding to the maximum acceptable output voltage of TENG from 4.3 to 25 kV, a fast response time of 10.2 ms, low pass filter with high gain of 40 dB/decade, and a reliable digital signal output has been developed. In addition, we further explore the universal applicability of the TSM for various working modes of TENG, the outputs generated from the TSM demonstrate good followability, and timely response, realizing the normalization of TENG output signals under different working modes. Finally, a smart switching system is constructed by integrating the TSM with a CS Mode TENG under the assistance of IoT platform technology, successfully realizing the comprehensive monitoring of the state of people at home and ensuring their security as well as the appliances safety. Such design of a universal energy solution for triboelectric sensors is applicable for managing and normalizing the outputs generated from various working modes of TENGs and suitable for facile integration with IoT platform, representing a significant step toward scaling up TENG applications in future smart sensing.

## 4 Experimental Section

### Design of Triboelectric Sensor Kit and Simulation

Circuit design software Multisim 14 launched by National Instruments Co., Ltd. and SPICE simulation were utilized to carry out the circuit design and simulation based on the impedance analysis theory (Part S1 and Theory S1, Supporting Information). The generic TSM mainly consisted of four modules including an adjustable input impedance voltage follower, an active low‐pass filter, voltage comparison, and indicator module, respectively. The adjustable input impedance voltage follower^[^ ^38^ ^]^ composed of an integrated operational amplifier (NE5532P), a potentiometer (2 MΩ), a resistor (100 KΩ), and a capacitor (1 µF) to realize the impedance matching function. The active low‐pass filter (first‐order) composed of an integrated operational amplifier (NE5532P), a resistor with resistance of 10 KΩ, and a capacitor with capacitance of 1 µF to realize filtering function. The voltage comparison function was implemented by a voltage comparison circuit consisting of a voltage comparator (LM393DR2G), a resistor, and a capacitor with capacitance of 1 µF, respectively, detailed parameters can be found in Table S3, Supporting Information. The indicator module was composed of *N*‐channel field effect transistor (BSS138), a resistor with resistance of 1 KΩ, and a LED bulb to realize the signal indication function.

The input and output characteristics, the voltage waveform, and amplitude of each functional node in the TSM were simulated and characterized through the software built‐in oscilloscope. The amplitude‐frequency and phase‐frequency characteristics were tested by the potter tester, and displayed in logarithmic form. The second and the third total harmonic distortion of TSM were examined by the distortion analyzer. The AC and DC voltage and current of each node were tested by a multi‐meter. After assembling and debugging of each instrument, the functions of step‐down, filtering, and voltage comparison for the TSM were well realized. The as‐designed TSM was subsequently drawn to the PCB diagram (Figure S7, Supporting Information) using Altium Designer software and printed out for further welding assembly.

### Circuit Calibration and Optimization

The TSM was calibrated by connecting to a 5 V power supply and keep away from the AC interference to observe the power indicator light was in the “Long‐on” state. An oscilloscope (MDO32, Tektronix) was utilized to observe the output waveform on each functional module to verify the design logic and calibrate the circuit. In the optimized TSM (Figure S8 and Table S4, Supporting Information), the integrated operational amplifier chip was replaced with TLV272ID, which had rail‐to‐rail output and larger input impedance. The filter circuit module was modified from an ordinary first‐order to a second‐order low‐pass active filter circuit, four different kinds of filters including the Butterworth, Chebyshev, Bessel, and Elliptic filter were selected and simulated by the MATLAB software as shown in Figure S9, Supporting Information. The general single‐limit comparator circuit was modified to a hysteresis comparator circuit,^[^ ^38^ ^]^ and the integration of the PCB board was also improved to raise the space utilization rate.

### Setup of Smart Switching System

The system was implemented by the internal program mounted on the ESP32 chip combined with the WiFi positioning, data storage and data visualization functions of the OneNET IoT platform. Among them, the internal program structure could be divided into three parts, including the function program, main program, and global static area variables. The program function could be divided into the signal reading and peripheral control part, data calculation part, state machine part, and network transmission part based on hypertext transfer protocol transmission protocol. For the reading and peripheral control part, it realized interaction with external devices including the reading of digital and analog signals as well as the control of switches on devices. The data calculation part realized the processing and calculation of data, including filtering and calculating the maximum and minimum values, and recording the number of presses. For the state machine part, it was responsible for recording and saving the state of the switch, including the single‐press, double‐press, triple‐press, and long‐press status. The network transmission protocol part was responsible for data upload and command, including uploading media access control address to use WiFi positioning service for calculating location, uploading device status, and cloud storage of voltage value read by TSM and data visualization services, as well as command distribution services for obtaining control device commands. The entire switch system used MCU with a main frequency of up to 40 MHz to ensure the real‐time signal processing performance, and applied the IoT platform service provided by China Mobile to improve the stability of remote control and communication, and realized a relatively stable intelligent switch internet function.

## Conflict of Interest

The authors declare no conflict of interest.

## Supporting information

Supporting Information

Supplemental Video 1

Supplemental Video 2

## Data Availability

The data that support the findings of this study are available on request from the corresponding author. The data are not publicly available due to privacy or ethical restrictions.
