# Supplementary material for: Universal Energy Solution for Triboelectric Sensors Toward the 5G Era and Internet of Things
Source: Adv Sci (Weinh). 2023 May 28;10(22):2302009. doi: 10.1002/advs.202302009 (PMC10401095; doi:10.1002/advs.202302009)
Supplement: Supplementary file 1 — Supporting Information [file ADVS-10-2302009-s003.pdf]

ADVANCED SCIENCE
Open Access

Supporting Information

for *Adv. Sci.*, DOI 10.1002/advs.202302009

Universal Energy Solution for Triboelectric Sensors Toward the 5G Era and Internet of Things

Haiyang Wen, Xiya Yang, Ruiyuan Huang, Duo Zheng, Jingbo Yuan, Hongxin Hong, Jialong Duan, Yunlong Zi and Qunwei Tang**

Supplementary

Universal energy solution for triboelectric sensors towards the 5G era and Internet of Things

Haiyang Wen, Xiya Yang, Ruiyuan Huang, Duo Zheng, Jingbo Yuan, Hongxin Hong, Jialong Duan, Yunlong Zi, and Qunwei Tang **

H. Wen, Prof. X. Yang, R. Huang, D. Zheng, J. Yuan, Dr. H. Hong, Prof. Q. Tang.

Institute of New Energy Technology

College of Information Science and Technology

Jinan University

Guangzhou 510632, PR China;

E-mail: xiyayang@jnu.edu.cn

Dr. H. Hong

School of Physics and Optoelectronics

South China University of Technology

Guangzhou, 510641, China;

Prof. J. Duan, Prof. Q. Tang.

Institute of Carbon Neutrality

College of Chemical and Biological Engineering

Shandong University of Science and Technology

Qingdao, 266590, China;

E-mail: tangqunwei@jnu.edu.cn

Prof. Y. Zi

Thrust of Sustainable Energy and Environment

The Hong Kong University of Science and Technology (Guangzhou)
Nansha, Guangzhou, Guangdong 511400, China;

Part 1. Circuit composition and functional circuit module

The voltage follower module is composed of an integrated operational amplifier to obtain sufficient voltage through a large input impedance and output through a low output impedance to realize the impedance conversion. In the voltage follower module, the DC bias circuit is composed of two large resistors and a variable resistor potentiometer to increase the static voltage from 0 to ~2.5 V to avoid distortion of the AC signal generated by the device before entering the circuit. Meanwhile, a 2MΩ variable resistor potentiometer is connected in parallel with the voltage follower module to adjust the input impedance of the entire circuit towards impedance matching, so that the voltage generated from the device can be adjusted when the original V_{oc} is in a wide range.

The first-order active filter module in the original TSM is a first-order active filter circuit, which consists of a resistor, a capacitor, and an integrated operational amplifier. The main function is to filter out high-frequency clutter to obtain low-frequency signals. The passband cutoff frequency can be calculated by:

$$f_p = \frac{1}{2\pi RC} \qquad Equation\ \mathrm{S1}$$

where f_p is the upper limit cutoff frequency, R is the resistance of R_4 and C is the capacitance of C_3.

The voltage magnification of the circuit can be given as:

$$\dot{A}_u = \frac{\dot{U}_o}{\dot{U}_i} = \frac{1}{1 + j\omega RC} = \frac{1}{1 + j\frac{f}{f_p}} \qquad Equation\ \mathrm{S2}$$

where f_p is the upper limit cutoff frequency and f is the current signal frequency.

In the optimized TSM, the filter module is a second-order active filter circuit consisting of three resistors, two capacitors and an integrated operational amplifier, which behaves better high-frequency filtering capability. The passband cutoff frequency for the optimized TSM can be calculated by:

$$f_p = \frac{1}{2\pi RC\sqrt{R\times C\times 10^3}} \qquad Equation\ \text{S3}$$

where R is the resistance value of $R_4 = R_5$, and C is the capacitance value of $C_3 = C_4$.

The voltage comparator module consists of two single-limit comparator circuits for each composing of a voltage comparator and two voltage divider resistors. The circuit function can be obtained by adjusting the resistance to change the threshold value of the comparison voltage. The threshold value can be calculated as:

$$V_{TH} = \frac{R_{D1}}{R_{D1} + R_{D2}} V_{CC} \qquad Equation\ \text{S4}$$

$$V_{TL} = \frac{R_{D3}}{R_{D3} + R_{D4}} V_{CC} \qquad Equation\ \text{S5}$$

where R_{D1} is the resistance value of the voltage divider resistor $R7$, R_{D2}, R_{D3}, R_{D4} are $R8$, $R9$ and $R10$, respectively. The comparator circuit outputs a stable high and low level after comparing the amplitude of the signal, and outputs a high level after the voltage rises and falls to a certain threshold. The signal indicator circuit will drive the LED to light up when the comparator circuit has a signal output.

In the third part of the original TSM, the voltage comparator module consists of two in-phase hysteresis comparator circuits (the transmission characteristics of the single-limit comparator and the hysteresis comparator are demonstrated in Supplementary **Figure S1** a-b). Each non-inverting hysteresis comparator circuit consists of a voltage comparator, a feedback resistor and two voltage

divider resistors, the threshold value of the comparison voltage is changed by adjusting the resistor value. According to *Eqn.* S6 to S8, the voltage divider resistance can be adjusted to modify the voltage divider size in the voltage comparator circuit to change the value of the reference voltage V_{Ref}, thereby changing the overall voltage judgment height of the two threshold values. The hysteresis difference between the two thresholds can be regulated through changing the feedback resistor R_f as given below (Note: there are four thresholds in total):

$$V_{TH} = \frac{R_i}{R_f}\left(V_{Ref} - V_{OL}\right) + V_{Ref} \qquad \textit{Equation}\ \text{S6}$$

$$V_{TL} = \frac{R_i}{R_f}\left(V_{Ref} - V_{OH}\right) + V_{Ref} \qquad \textit{Equation}\ \text{S7}$$

$$V_{Ref} = V_{CC} \times \frac{R_{d1}}{R_{d1}+R_{d2}} \qquad \textit{Equation}\ \text{S8}$$

where R_{D1} is the voltage dividing resistor $R10/R12$, R_{D2} is the voltage dividing resistor $R11/R13$, V_{Ref} is the reference voltage value, R_f is the feedback resistor $R14/R15$, R_i is the input resistor $R8$, V_{OH} and V_{OL} are the high and low output voltage of the voltage comparator, respectively.

Theory S1. Impedance Theory Analysis

TENG's electrical output simulation model can be equivalent to a model composed of a voltage source connected in series with a small-capacity capacitor[1]. It is precisely because of the high output impedance brought by the capacitance effect that impedance matching as an important link most important part of TENG's signal management circuit, which should be considered and resolved at first. The impedance of the capacitor in the TENG electrical output equivalent model can be calculated by the following formula:

$$Z_c = \frac{1}{2\pi fc} \qquad Equation\ \text{S9}$$

Among them, Z_c is the equivalent impedance of the equivalent capacitance of TENG, in Ω; f is the frequency of the signal generated by TENG, in Hz; c is the size of the equivalent capacitance of TENG, in F.

According to Ohm's law, the voltage divided by the load after the internal resistance and the load is shown in the following formula:

$$V_l = \frac{R_l}{R_s + R_l} \times V_s \qquad Equation\ \text{S10}$$

Among them, V_l is the external load voltage of the TENG equivalent circuit in V; R_s is the internal impedance of the TENG equivalent circuit, in Ω; R_l is the load resistance of the TENG equivalent circuit, in Ω; V_s is TENG the original voltage value generated, in V.

In order to solve this problem, it is necessary to select a device or circuit with a higher input impedance to match the high output impedance of TENG. Among the active devices, the integrated operational amplifier, as an active device widely used in analog circuits, adopts a common-collection amplifier circuit, which is suitable for the signal processing circuit of TENG.

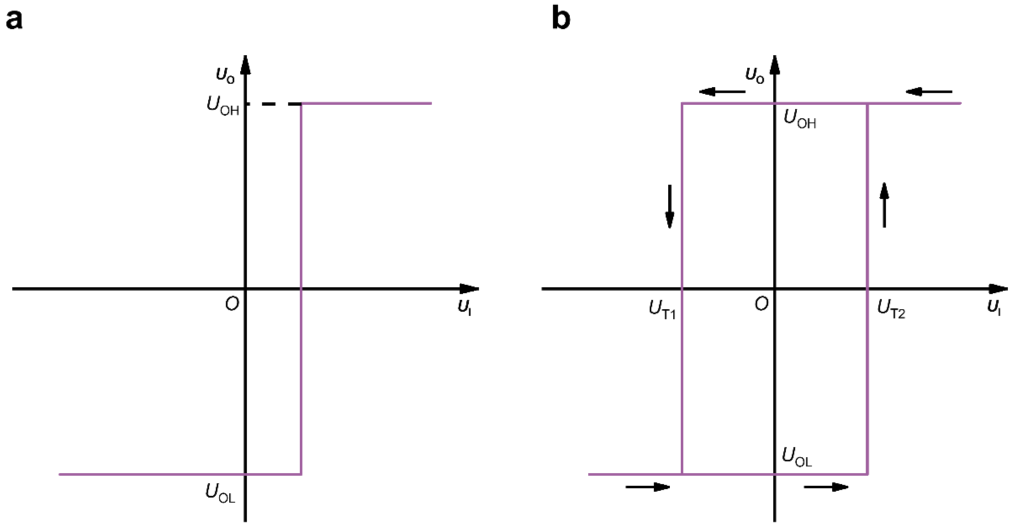

Figure S1. Voltage transfer characteristics of two kinds of comparators. (a) Characteristics of general single-limit comparators. (b) Characteristics of general hysteretic comparator.

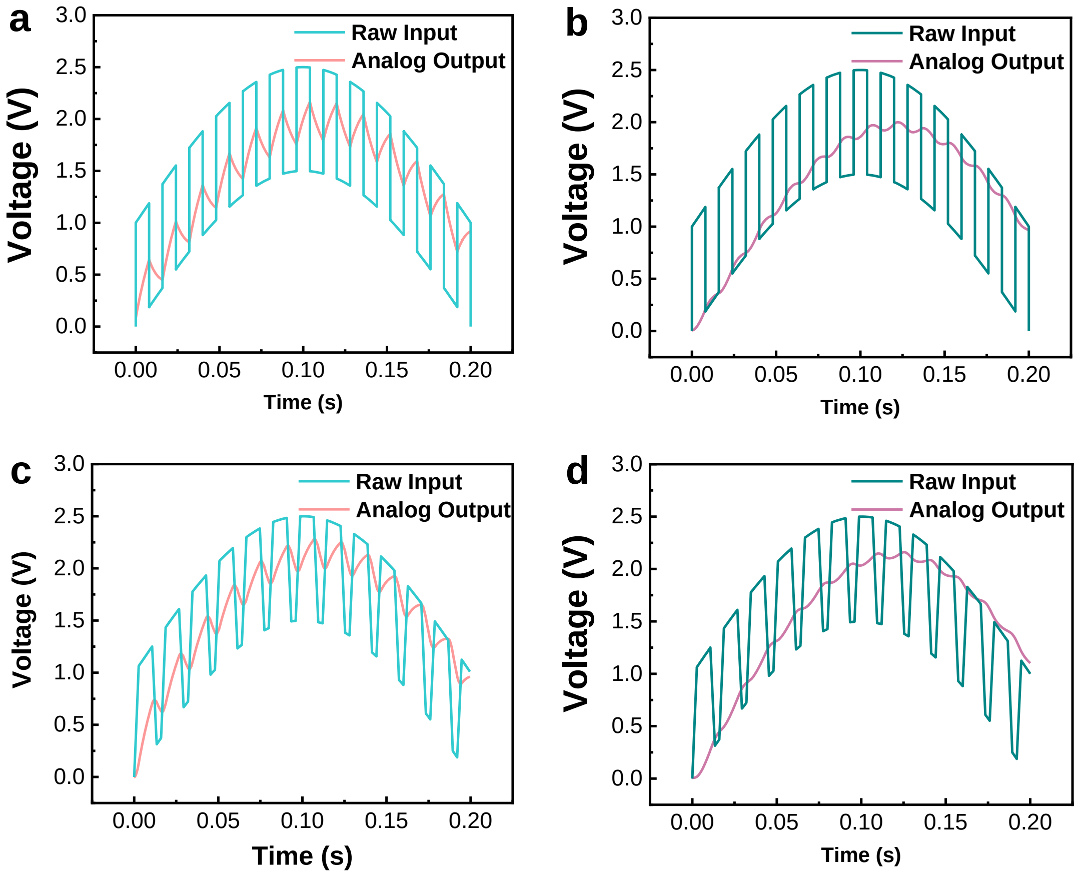

Figure S2. Interference wave simulation of two versions of the circuit. (a,b) Rectangular interference wave simulation of original and optimized TSM. (b,d) Triangular interference wave simulation of original and optimized TSM.

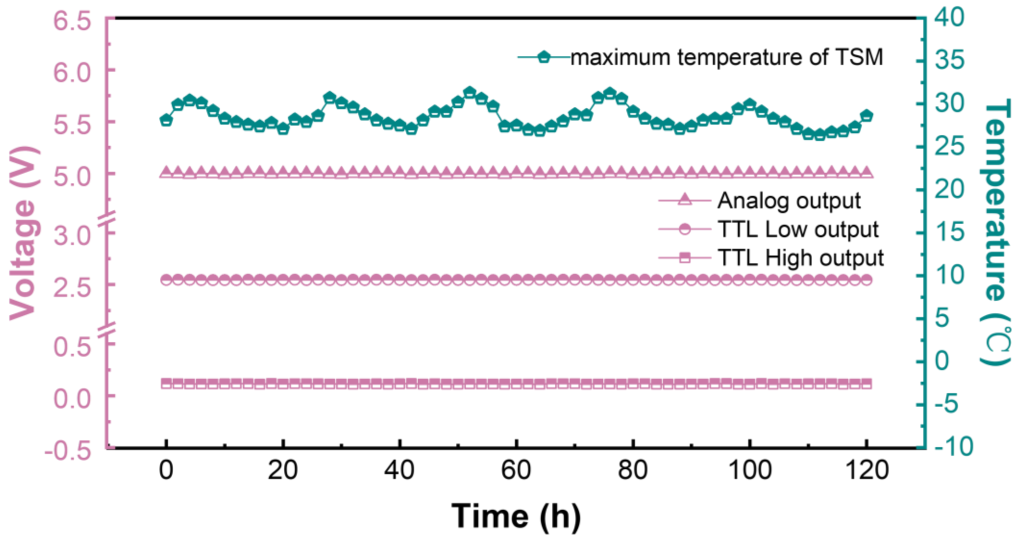

Figure S3. Working stability test of TSM within 5 days.

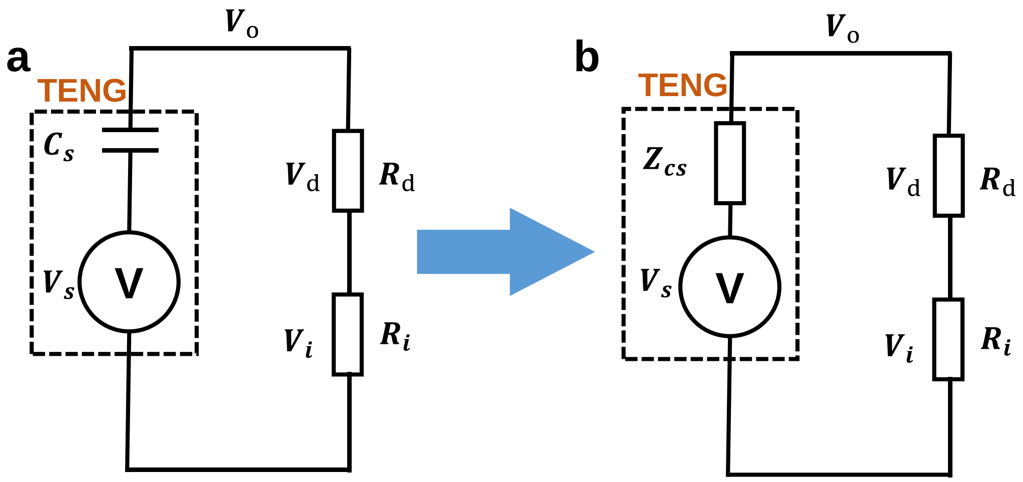

Figure S4. Equivalent circuit conversion of the test circuit. (a) Original equivalent circuit. (b) Pure resistance equivalent circuit.

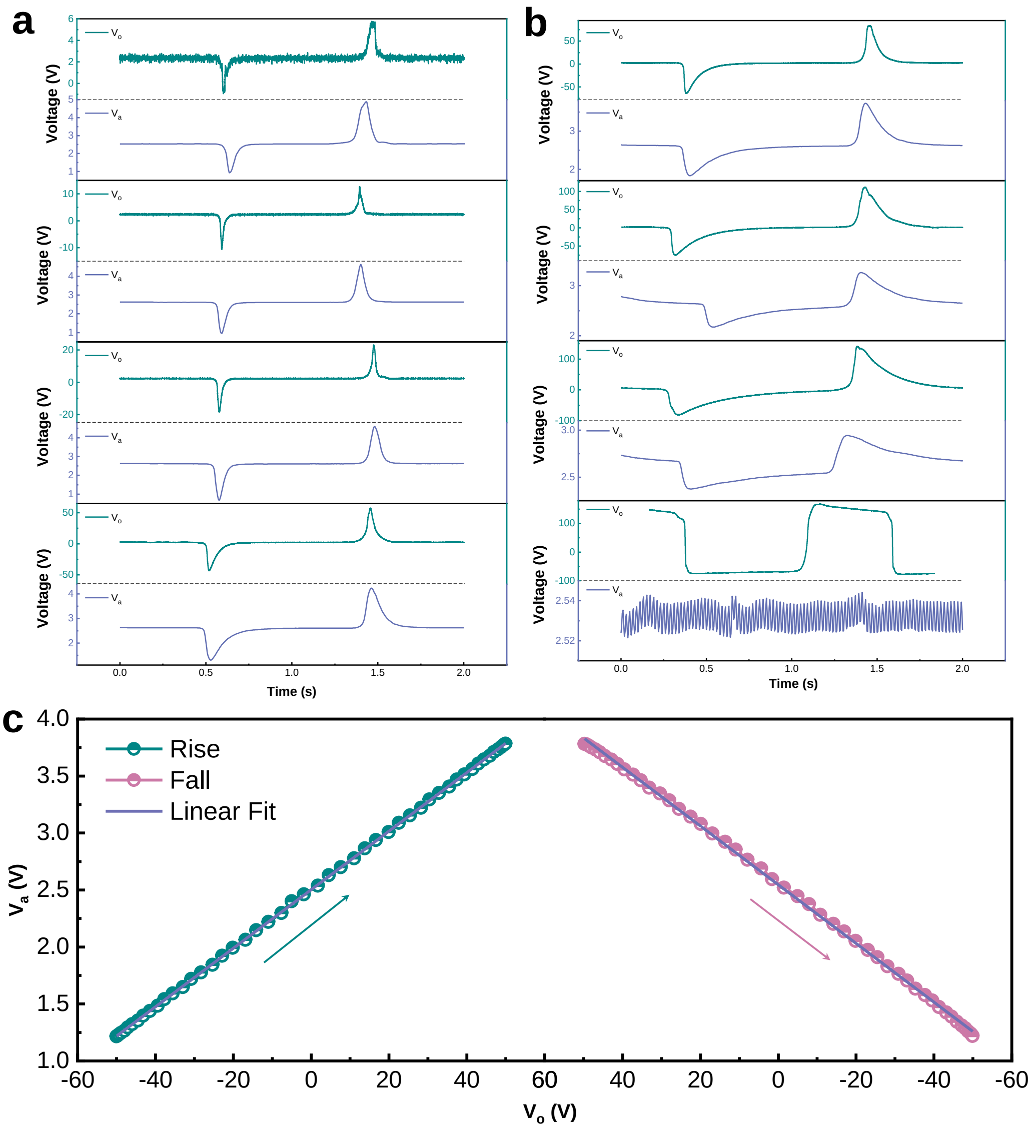

Figure S5. Combination test of TENG and circuit. (a, b) Correspondence between TENG waveform and circuit waveform with resistance change. (c) Corresponding linear relationship simulation data of TENG output and circuit output.

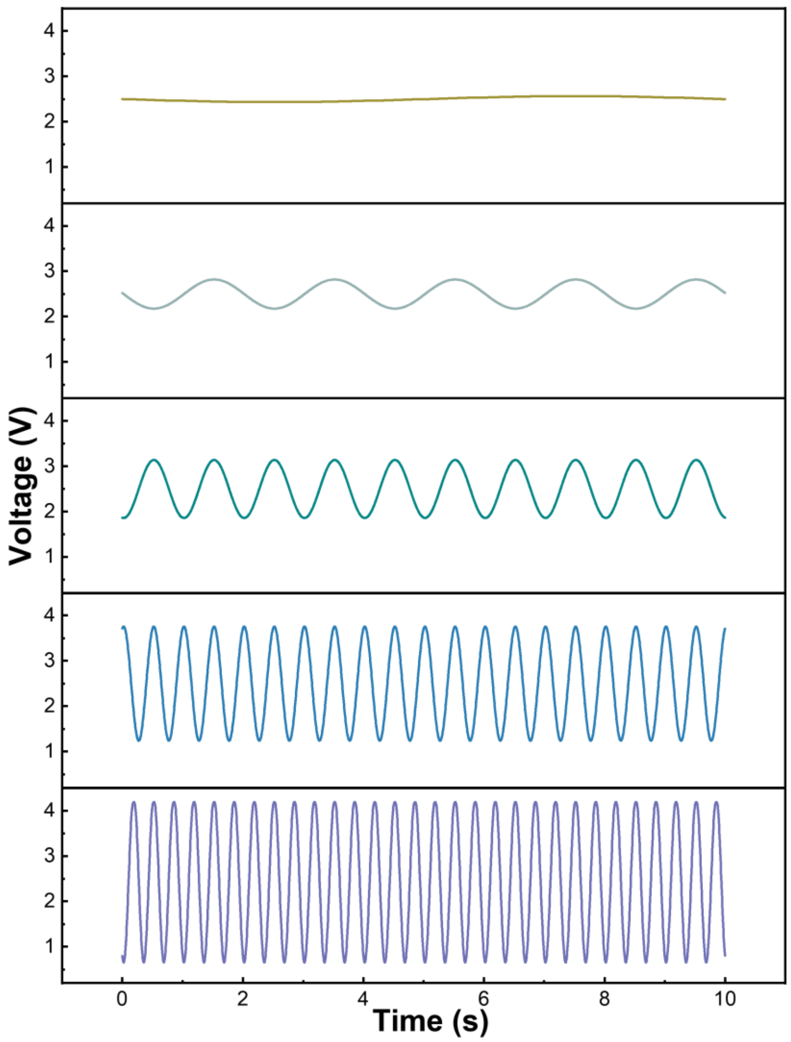

Figure S6. The waveform of circuit output as change of TENG frequency in simulation.

a

b

c

d

e

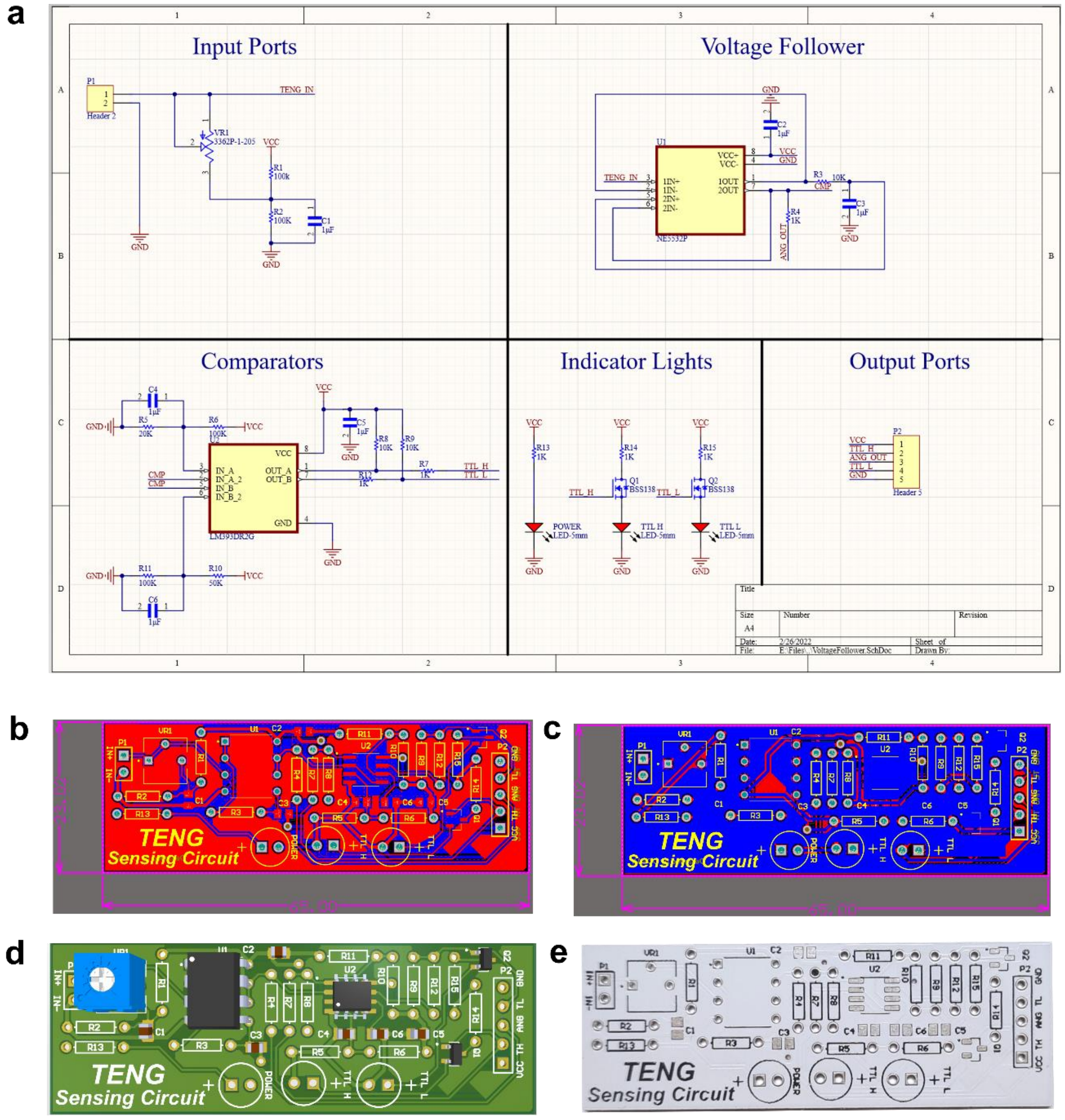

Figure S7. Electrical diagram of the original TSM. (a) Circuit schematic diagram. (b-e) PCB diagram.

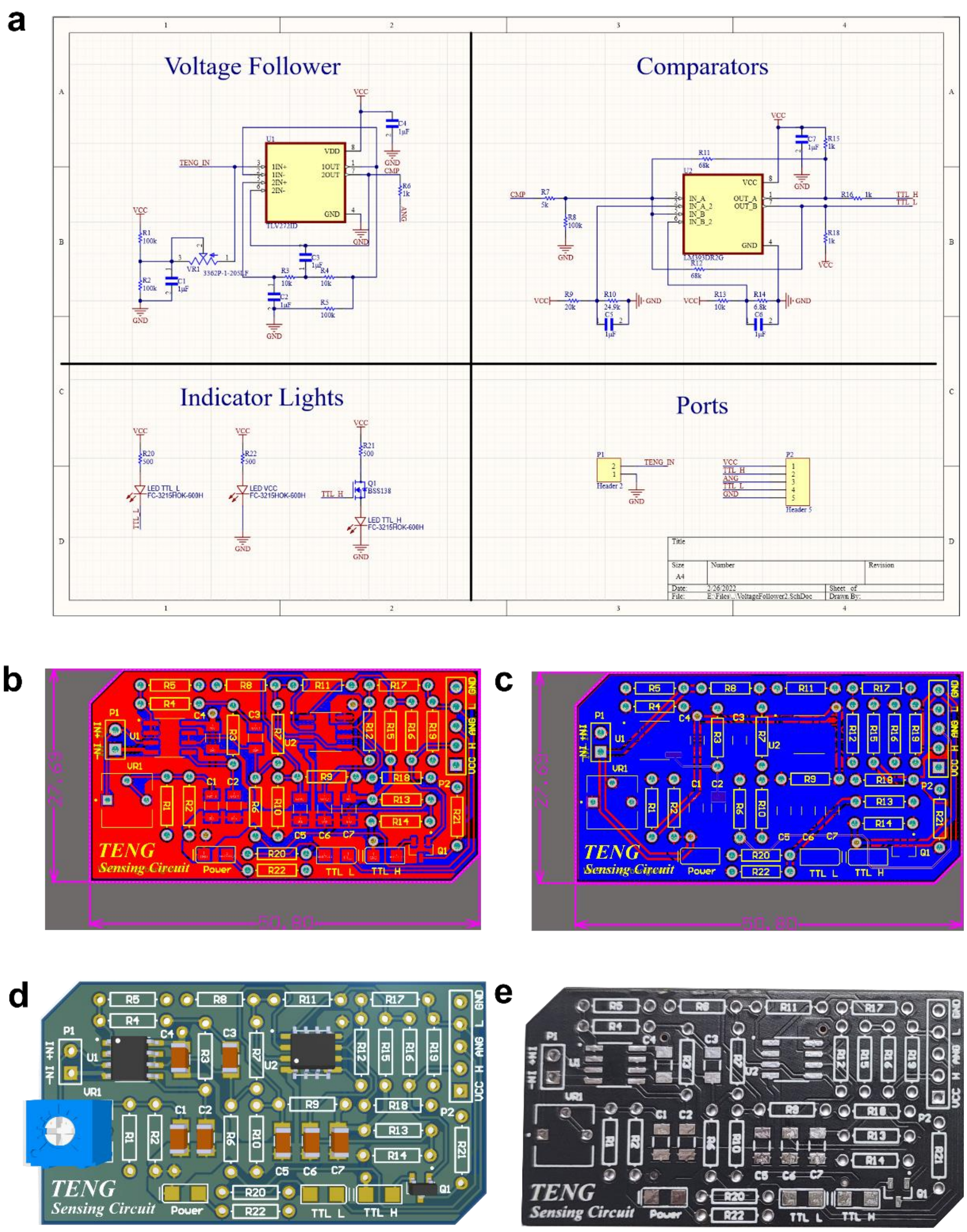

Figure S8. Electrical diagram of the optimized TSM. (a) Circuit schematic diagram. (b-e) PCB diagram.

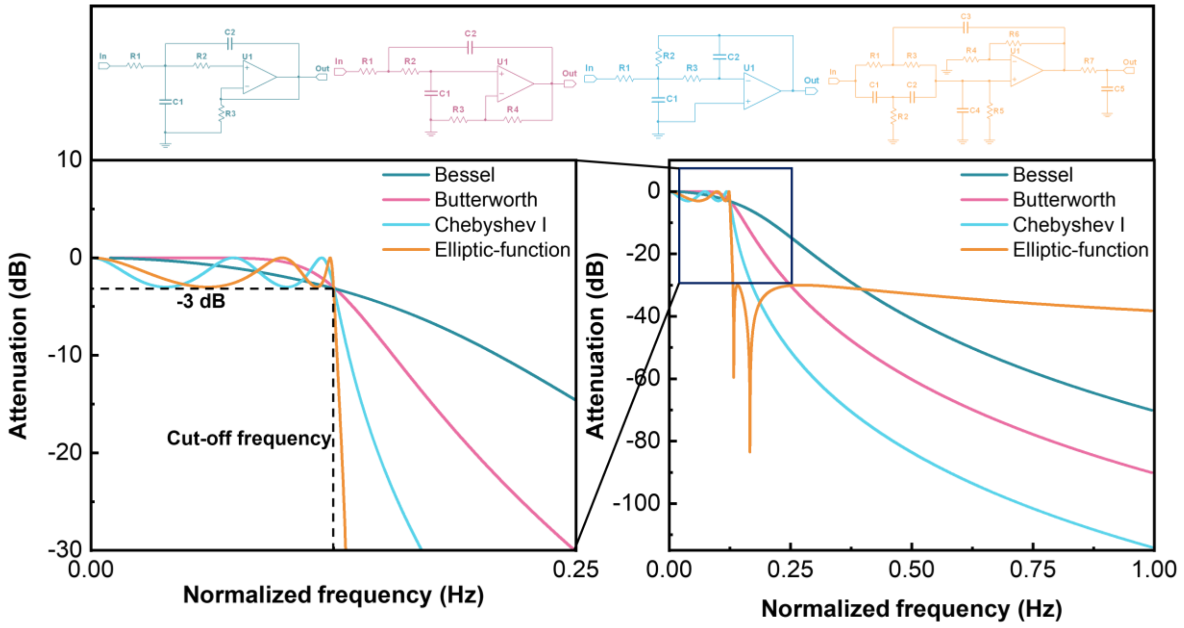

Figure S9. Simulation diagram of amplitude-frequency characteristics of four different filter circuits.

Table S1. Comparison of various functional parameters of peers' works.

Parameters \ References	[2]	[3]	[4]	[5]	[6]	[7]	This work
Voltage bias	No	Yes	No	No	No	Yes	Yes
Input impedance adjustment	No	No	No	No	No	No	Yes
Acceptable V_{OC} range of TENG	--	--	--	--	--	--	0-8 kV (5 V supply)
Filtering ability	Yes	Yes	No	No	No	No	Yes
Analog output	Yes	Yes	Yes	Yes	No	No	Yes
Digital conversion capability	No	No	No	No	Yes	Yes	Yes
Response time	--	--	--	--	8 ms	--	10.2 ms
Power consumption	--	--	--	--	--	--	3.93 mW (5 V supply)

Table S2. Electrical output performance parameters for the optimized TSM.

PARAMETER	DESCRIPTION	TEST CONDITIONS	VALUE	UNIT	REMARK
V_{CC}	Supply voltage		2.7~16	V	Typical: 3.3 V, 5 V
R_i	Input resistance	25℃, Supply voltage: V_{CC}=5V, V_{EE}=0V	0~2	mΩ	Adjustable
R_{oa}	Output resistance		0.04	Ω	
R_{ol}			0.02		
R_{oh}			0.25		
f_H	Cut-off frequency		10.23	Hz	Adjustable
V_I	Input bias voltage		2.09	V	
V_{OA}	Output bias voltage		2.54		
V_{OL}			4.99		
V_{OH}			0.21		
V_{TH}	Upper threshold voltage		2.96		Adjustable
			2.60		
V_{TL}	Lower threshold voltage		2.46		
			2.10		
V_{oa}	Output voltage range		0~4.66		
V_{ol}			4.99, 0.30		
V_{oh}			0.21, 4.98		
A_a	Attenuation		40	dB/Decade	
t_R	Response time		10.2	ms	

Table S3. Component parameters and models of the original TSM.

<table>
<tr><th>PARAMETER</th><th>VALUE</th><th>UNIT</th><th>MODEL</th><th>MANUFACTURER</th><th>REMARK</th></tr>
<tr><td>R1</td><td>2</td><td>MΩ</td><td>3362P-1-205LF</td><td>BOURNS</td><td>Potentiometer</td></tr>
<tr><td>R2,R3,R7,R10</td><td>100</td><td rowspan="5">kΩ</td><td>MFR0W4F1003A50</td><td>UNI-ROYAL</td><td>--</td></tr>
<tr><td>R9</td><td>50</td><td>MF1/4W ±1% 50K6 STB5</td><td>VO</td><td>--</td></tr>
<tr><td>R8</td><td>20</td><td>MFR0W4F2002A50</td><td rowspan="2">UNI-ROYAL</td><td>--</td></tr>
<tr><td>R4,R11,R12</td><td>10</td><td>MFR0W4F1002A50</td><td>--</td></tr>
<tr><td>R5,R6,R13,R14,R15,R16,R17,R18</td><td>1</td><td>RN 1/4W 1K F T/B A1</td><td>Tyohm</td><td>--</td></tr>
<tr><td>C1~C6</td><td>1</td><td>μF</td><td>CL21B105KPFNNNE</td><td>SAMSUNG</td><td>--</td></tr>
<tr><td>U1A,U1B</td><td>--</td><td>--</td><td>NE5532P</td><td>TI</td><td>Amplifier</td></tr>
<tr><td>U2A,U2B</td><td>--</td><td>--</td><td>LM393DR2G</td><td>onsemi</td><td>Comparator</td></tr>
<tr><td>Q1,Q2</td><td>--</td><td>--</td><td>BSS138</td><td>AnBon</td><td>N-MOS</td></tr>
</table>

Table S4. Component parameters and models of the optimized TSM.

PARAMETER	VALUE	UNIT	MODEL	MANUFACTURER	REMARK
R1	2	MΩ	3362P-1-205LF	BOURNS	Potentiometer
R2,R3,R6,R7,R9	100	kΩ	MFR0W4F1003A50	UNI-ROYAL	--
R14,R15	68		MFR0W4F6802A50		--
R13	24.9		RN1/2WS24.9KΩFT/BA1	Tyohm	--
R12	20		MFR0W4F2002A50	UNI-ROYAL	--
R4,R5,R10	10		RN1/2WS24.9KΩFT/BA1	Tyohm	--
R11	6.8		RN1/2WS6.04KΩFT/BA1		--
R8	5.1		MF1/4W-5.1KΩ±1%T52	Huaxing Electromechanical Co., Ltd.	--
R16,R17,R18,R19,R20,R21,R22,R23	1		MFR0W4F1001A50	UNI-ROYAL	--
C1~C7	1	μF	CL31A105KB9LNNC	SAMSUNG	--
U1A,U1B	--	--	TLV272ID	TI	Amplifier
U2A,U2B	--	--	LM393DR2G	onsemi	Comparator
Q1	--	--	BSS138	CJ	N-MOS
LED	--	--	XL-3216UOC	XINGLIGHT	--

References

[1] S. M. Niu, Y. S. Zhou, S. H. Wang, Y. Liu, L. Lin, Y. Bando, Z. L. Wang, *Nano Energy* **2014**, 8, 150.

[2] Y. Luo, Z. H. Wang, J. Y. Wang, X. Xiao, Q. Li, W. B. Ding, H. Y. Fu, *Nano Energy* **2021**, 89, 106330.

[3] M. L. Zhu, Z. D. Sun, C. K. Lee, *ACS Nano* **2022**, 16, 14097.

[4] S. Lu, W. Q. Lei, L. X. Gao, X. Chen, D. Q. Tong, P. F. Yuan, X. J. Mu, H. Yu, *Nano Energy* **2021**, 87, 106137.

[5] X. X. Shi, S. D. Zhang, S. Q. Gong, *J Mater Chem A* **2020**, 8, 8997.

[6] X. P. Chen, J. Y. Li, Y. N. Liu, J. X. Jiang, C. Zhao, C. Z. Zhao, E. G. Lim, X. H. Sun, Z. Wen, *ACS Appl Mater Inter* **2021**, 13, 61789.

[7] C. K. Qiu, F. Wu, Q. F. Shi, C. K. Lee, M. R. Yuce, *IEEE Access* **2019**, 7, 92745.
